# LncRNA XIST acts as a tumor suppressor in prostate cancer through sponging miR-23a to modulate RKIP expression

**DOI:** 10.18632/oncotarget.21719

**Published:** 2017-10-10

**Authors:** Yang Du, Xiao-Dong Weng, Lei Wang, Xiu-Heng Liu, Heng-Cheng Zhu, Jia Guo, Jin-Zhuo Ning, Cheng-Cheng Xiao

**Affiliations:** ^1^ Department of Urology, Ren’min Hospital of Wuhan University, Wuhan, Hubei 430000, China

**Keywords:** prostate cancer, long non-coding RNA, XIST, miR-23a, RKIP

## Abstract

Accumulating evidences have indicated that aberrant expression of long non-coding RNAs (LncRNAs) is tightly associated with cancer development. Previous studies have reported that lncRNA XIST regulates tumor malignancies in several cancers. However, the underlying mechanism of XIST in prostate cancer remains unclear. In the current study, we found that XIST was down-regulated in prostate cancer specimens and cell lines. Low expression of XIST was correlated with poor prognosis and advanced tumor stage in prostate cancer patients. In gain and loss of function assays, we confirmed that XIST suppressed cellular proliferation and metastasis in prostate cancer both *in vitro* and *in vivo*. Furthermore, we found that XIST negatively regulates the expression of miR-23a and subsequently promotes RKIP expression at post-transcriptional level. Consequently, we investigated the correlation between XIST and miR-23a, and identified miR-23a as a direct target of XIST. In addition, over-expression of miR-23a efficiently abrogated the up-regulation of RKIP induced by XIST, suggesting that XIST positively regulates the expression of RKIP by competitively binding to miR-23a. Taken together, our study indicated that lncRNA XIST acts as a tumor suppressor in prostate cancer, and this regulatory effect of XIST will shed new light on epigenetic diagnostics and therapeutics in prostate cancer.

## INTRODUCTION

Prostate cancer (PCa) is the most common malignancy in males, accounting for 15% of the cancers diagnosed in men and 13% of cancer-related deaths [1]. It has been elucidated that tumor growth in early-stage prostate cancer requires the presence of androgens [2]. Although androgen deprivation therapy (ADT) has been the main treatment used for androgen-dependent prostate cancer (ADPC) since 1940, tumors tend to relapse after a remission stage of approximately 18-24 months, and grow in an androgen-independent manner [3, 4]. However, the mechanism underlying androgen-independent prostate cancer (AIPC) development remains unclear, and AIPC related biomarkers are limited. Thus, it is vitally important for us to explore new, detailed signaling pathway in AIPC and determine novel effective molecular biomarkers.

As the human genome project has revealed, only 2% of our genome is protein-coding genes, while the rest of the genome is actively transcribed as non-coding RNAs (ncRNAs) [5]. Among them, long non-coding RNA (lncRNA), a newly discovered ncRNA, has provoked many concerns. LncRNAs are a class of non-encoding RNA transcripts with more than 200 nucleotides in length [6]. They were previously deemed as byproducts transcribed from RNA polymerase II without biological function [7]. Now, as recent studies have demonstrated, lncRNAs participate not only in cellular development and differentiation, but also in tumorigenesis [8, 9]. They can regulate gene expression in multiple ways, affecting processes such as chromatin structure, cutting and splicing, nuclear transport, transcriptional modification, RNA decay and epigenetic control [10–13]. Therefore, lncRNAs possess great potential as new therapeutic targets in cancer research.

Aberrant expression of lncRNAs has been observed in a variety of cancers. Increasing evidence has indicated that lncRNAs may function as tumor suppressors, oncogenes, or even both in different contexts [14]. For example, Braconi et al found that upregulation of lncRNA MEG3 correlates with carcinogenesis and poor prognosis in hepatocellular cancer [15]. Cui et al showed that lncRNA SNHG1 contributes to tumor progression in lung cancer through inhibition of miR-101-3p [16]. Li et al showed that lncRNA n340790 accelerates cell proliferation in thyroid cancer by targeting miR-1254 [17]. Zeng et al reported that lncRNA AF113014 acts as a tumor suppressor in hepatocellular carcinoma cells by promoting Egr2 expression [18].

In the present study, we confirmed that down-regulation of XIST is a characteristic molecular change in prostate cancer, and the expression of XIST is negatively correlated with clinical stage, metastasis and Gleason score. Low expression of XIST may lead to poor prognosis in prostate cancer patients. Function assays suggested that overexpression of XIST inhibited proliferation, migration and invasion in prostate cancer cells *in vitro* and *in vivo*. Upon further study, we predicted and identified miR-23a as a downstream target of XIST. In addition, RKIP was demonstrated to be a direct target of miR-23a and acted as a tumor suppressor in prostate cancer progression. Together, our work provides a new prospect in comprehending the role of lncRNA XIST in prostate cancer, and the regulatory effect of lncRNA XIST will shed new light on molecular diagnostics and therapeutics in prostate cancer.

## RESULTS

### LncRNA XIST is low expressed in prostate cancer tissues and cell lines

**Figure 1:**
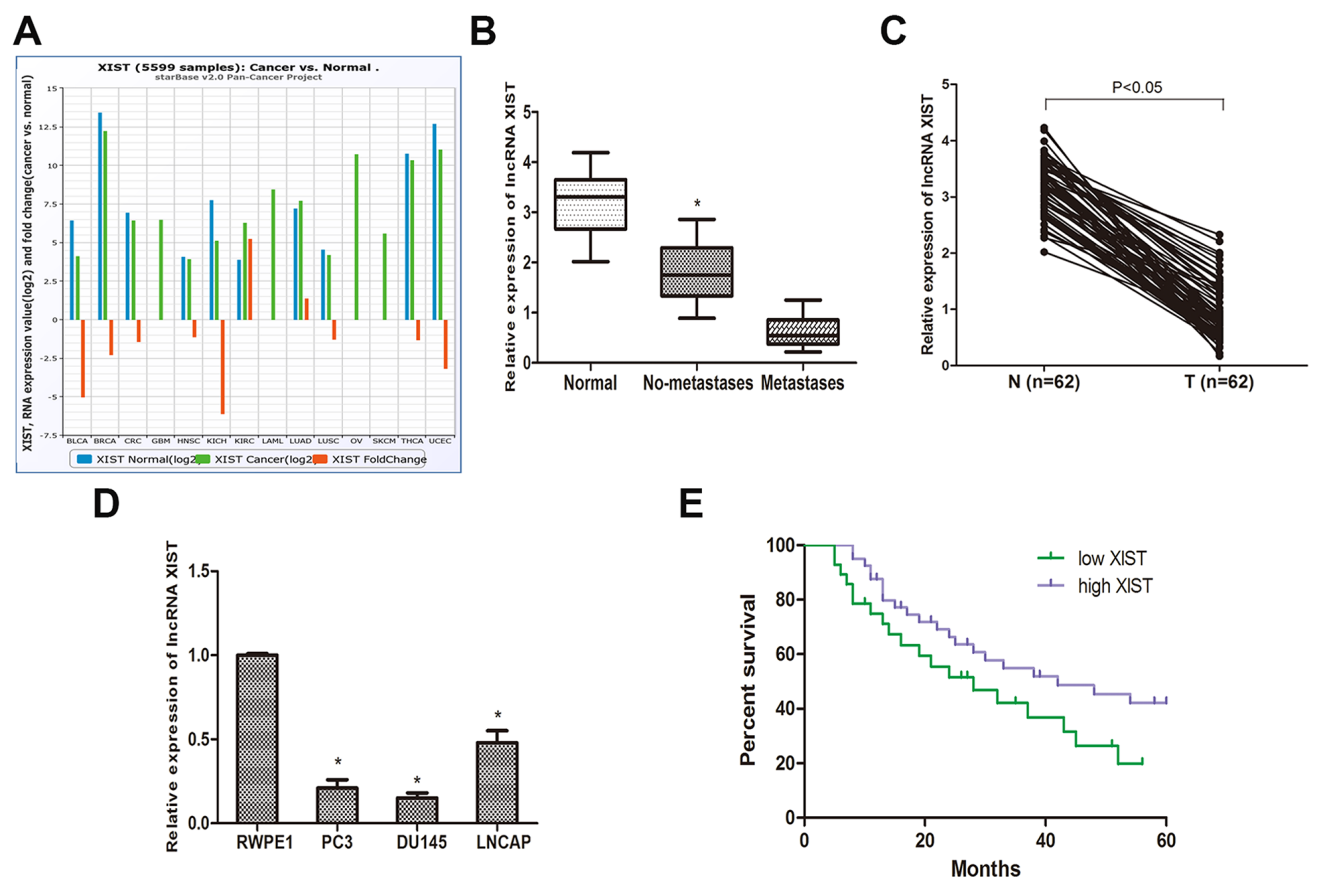
LncRNA XIST is low expressed in prostate cancer tissues and cell lines **(A)** Pan-Cancer analysis of XIST expression in 14 cancer types from The Cancer Genome Atlas (TCGA) Data Portal from starBASE v2.0. **(B)** The expression of XIST is negatively correlated with metastasis in prostate cancer specimens. *P<0.05 vs. Normal groups and metastases groups. **(C)** Relative expression of XIST in prostate cancer samples and adjacent normal prostate tissues. **(D)** QRT-PCR analysis of prostate cancer cell lines (PC3, DU145 and LNCAP) and human prostate epithelial cell line RWPE1. *P<0.05 compared with RWPE1 cells. **(E)** Kaplan-Meier analysis of the correlation between XIST expression and overall survival of patients with prostate cancer (The log-rank test was used to calculate P-values). *P<0.05 vs. low XIST expression group.

To determine the role of lncRNA XIST in different cancers, we explored the expression of XIST in the TCGA data portal from Starbase ver2.0. Pan-cancer analysis showed that XIST was down-regulated in a wide range of cancers (Figure 1A). Then, we determined the expression of XIST in prostate cancer specimens and adjacent normal tissues from 62 prostate cancer patients by qRT-PCR. The results revealed that XIST expression was significantly down-regulated in prostate cancer tissues compared to normal tissues (Figure 1C) and negatively correlated with the metastasis of prostate cancer (Figure 1B). In addition, we investigated the expression of XIST in the normal human prostate epithelial cell line RWPE1 and 3 types of human prostate cancer cell lines (PC3, DU145 and LNCAP). We found that the expression of XIST was considerably reduced in the 3 prostate cancer cell lines compared to RWPE1 (Figure 1D). Collectively, these data suggest that XIST may be involved in the occurrence and development of prostate cancer.

### Low expression of XIST is correlated with poor outcomes in prostate cancer patients

To better understand the correlation between lncRNA XIST and clinical features of prostate cancer patients, we analyzed clinical data that we collected from 62 prostate cancer patients. Based on the cut-off of ROC curve, we separated all patients into two groups. The data revealed that the expression of XIST was highly correlated to the clinical stage, metastasis, Gleason score and preoperative PSA level (Table 1, P<0.05). However, there was no apparent association between XIST expression and other clinical features, such like age (Table 1, P>0.05). We then investigated whether XIST expression is correlated with the overall survival time in prostate cancer patients. A Kaplan-Meier analysis and log-rank test were performed, and the results showed that patients with low expression of XIST had a poor overall survival time compared to patients with high XIST expression (Figure 1E).

**Table 1:** Correlation of relative lncRNA XIST expression with the clinicopathological characteristics of patients with prostate cancer

	LncRNA XIST expression^*^				
Parameters	Group	High	Low	Total	P value
Age	<70	18	9	27	0.324
	≥70	19	16	35	
Metastasis	Absence	26	7	33	<0.01
	Presence	11	18	29	
Clinical stage	T1	20	5	25	0.012
	T2/T3	18	19	37	
Preoperative PSA	<4	14	4	18	0.044
	4-10	6	7	13	
	>10	13	18	31	
Gleason score	<7	17	5	22	0.019
	7	4	6	10	
	>7	12	18	30	

*The mean (2.2) of the relative expression of lncRNA XIST in tumor tissues of all paired samples was used as a cut-off to classify a tumor sample was high or low according to their XIST expression levels.

### Influence of lncRNA XIST on the malignant phenotypes of prostate cancer *in vitro*

**Figure 2:**
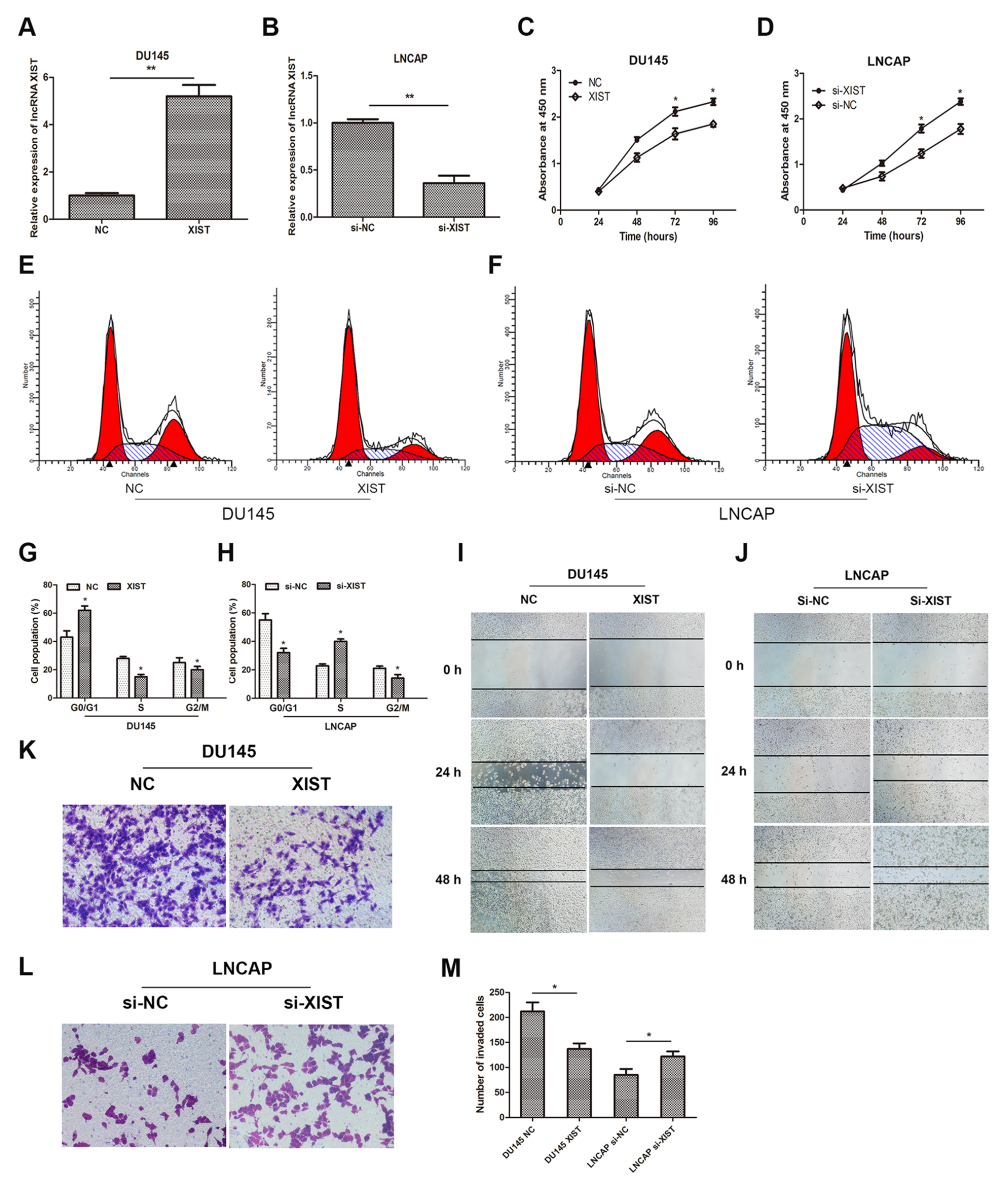
Influence of lncRNA XIST on the malignant phenotypes of prostate cancer *in vitro* **(A, B)** Relative expression of XIST in DU145 or LNCAP cells after transfection with XIST or si-XIST. **P<0.01 vs. NC group or si-NC group. **(C, D)** CCK-8 assays were performed to determine cellular proliferation in DU145 cells transfected with XIST and LNCAP cells transfected with si-XIST. *P<0.05 vs. NC group or si-NC group. **(E, G)** Cell cycle of DU145 cells transfected with XIST or NC was detected by flow cytometry. *P<0.05 vs. NC group. **(F, H)** Cell cycle of LNCAP cells transfected with si-XIST or si-NC was examined by flow cytometry. *P<0.05 vs. si-NC group. **(I, J)** Wound healing assays were performed to evaluate cell migratory ability in DU145 cells transfected with XIST or NC and LNCAP cells transfected with si-XIST or si-NC. **(K-M)** Transwell invasion assays were performed to examine invasive ability in cells. Cell number was counted in five random fields at 200 × magnification. *P<0.05 vs. NC group or si-NC group. Data are presented as mean ± SD from three separated experiments.

To investigate the biological functions of lncRNA XIST in prostate cancer cells, we increased expression of XIST in DU145 cells by transfection of recombinant adenoviruses and suppressed that in LNCAP cells by small interfering RNA (siRNA). QRT-PCR was employed to verify the transfection effect, and the results suggested that expression of XIST was remarkably upregulated in adenovirus-XIST transfected DU145 cells and was significantly suppressed in si-XIST transfected LNCAP cells, compared with negative control groups (Figure 2A, 2B). In addition, data from CCK-8 assay showed that over-expression of XIST remarkably suppressed cellular viability of DU145 cells compared to control groups, whereas suppression of XIST moderately promoted cell growth in LNCAP cells (Figure 2C, 2D). Cell cycle assay was also performed to evaluate the effect of XIST on cell proliferation. Our data showed that over-expressed XIST markedly impeded cell proliferation in DU145 cells at G1/G0 phase (Figure 2E, 2G), while knockdown of XIST significantly accelerated that in LNCAP cells at S phase (Figure 2F, 2H). Furthermore, we explored whether XIST was involved in cell migration and invasion in DU145 and LNCAP cell lines. As the data of wound healing assay showed that, over-expression of XIST remarkably mitigated cell migration in DU145 cells (Figure 2I). However, knockdown of XIST caused an opposite result in contrast (Figure 2J). By using cell invasion assay, we observed that the number of invaded cells was obviously decreased in the XIST over-expressing cells compared with control groups, while that was markedly increased in XIST-silencing cells (Figure 2K, 2L, 2M). Taken together, these findings indicated that over-expression of XIST inhibited cell proliferation, migration and invasion in prostate cancer cells.

### LncRNA XIST inhibits miR-23a expression by directly targeting it

It has been elucidated that lncRNAs contain complementary sequences to miRNA. Based on this ground, they can competitively binding to miRNAs and function as a competing endogenous RNAs (ceRNAs). Therefore, we asked whether lncRNA XIST has a similar function in prostate cancer.

**Figure 3:**
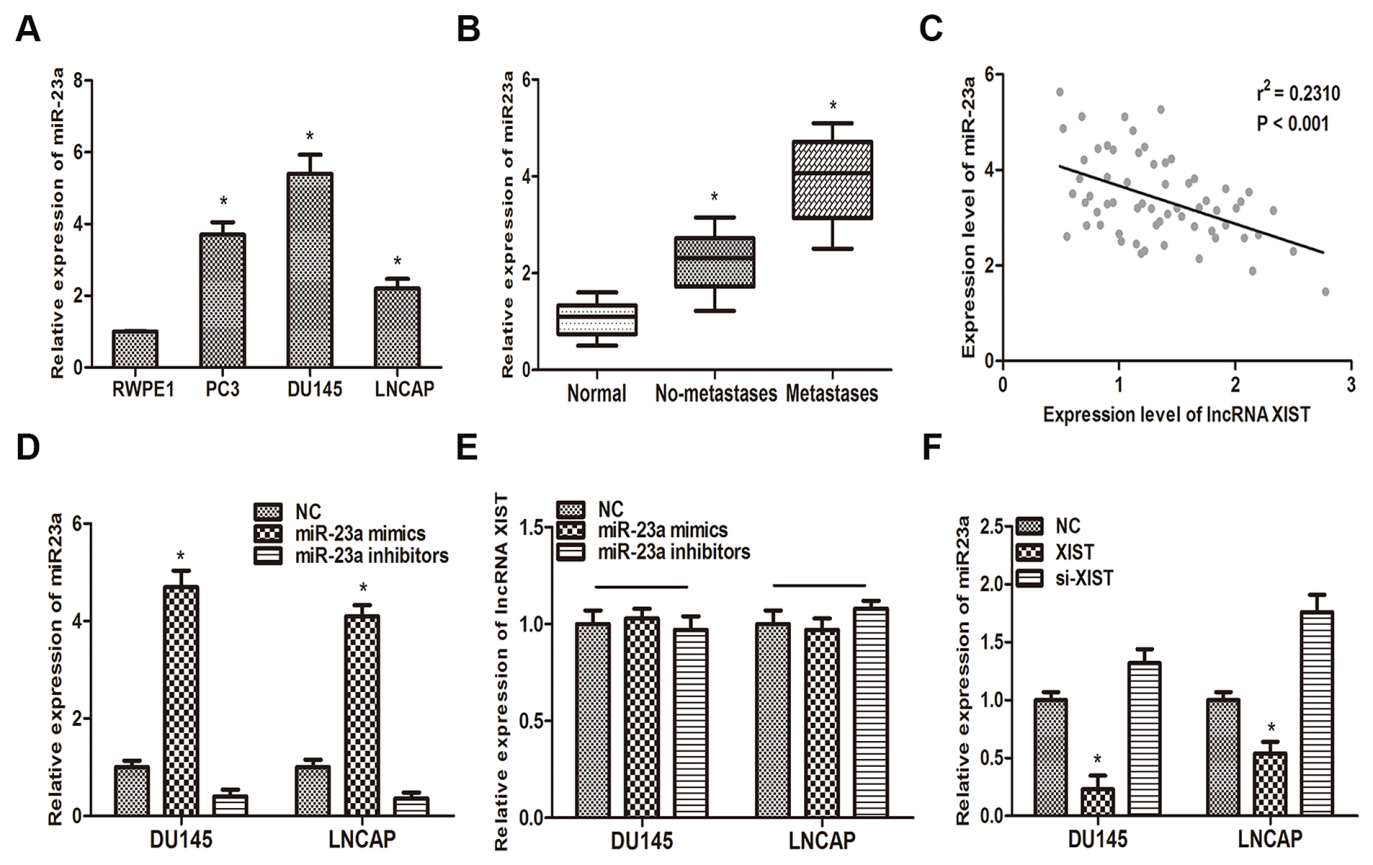
MiR-23a is inversely correlated with the expression of XIST **(A)** MiR-23a expression in prostate cancer cell lines (PC3, DU145 and LNCAP) and human prostate epithelial cell line RWPE1 was examined by qRT-PCR. *P<0.05 vs. RWPE1 group. **(B)** miR-23a expression was negatively correlated with metastasis in prostate cancer. *P<0.05 vs. Normal group. **(C)** Pearson’s correlation analysis was performed to determine the relationship between miR-23a and lncRNA XIST. **(D)** The expression level of miR-23a in DU145 and LNCAP cells transfected with miR-23a mimics and miR-23a inhibitors was measured by qRT-PCR. *P<0.05 vs. respective control group. **(E)** Relative expression of XIST in DU145 and LNCAP transfected with miR-23a mimics and miR-23a inhibitors was determined by qRT-PCR. **(F)** Relative expression of miR-23a in DU145 and LNCAP cells transfected with XIST and si-XIST was examined by qRT-PCR. *P<0.05 vs. respective control group. Data are presented as mean ± SD in three independent experiments.

Previous study has reported that miR-23a played a key role in metastasis of prostate cancer [19]. In this context, we reexamined the correlation between miR-23a and prostate cancer. QRT-PCR analysis suggested that miR-23a was highly upregulated in the three prostate cancer cell lines (PC3, DU145, LNCAP), compared with normal prostate epithelial cell lines (RWPE1) (Figure 3A). Furthermore, our findings suggested that miR-23a is highly expressed in metastatic prostate cancer tissues compared with non-metastatic tumor tissues and paired normal tissues (Figure 3B). Then, a Pearson’s correlation analysis was performed. Our data suggested that miR-23a expression level was negatively associated with the expression of XIST in 62 prostate cancer patients (Figure 3C).

To further delineate the interaction between lncRNA XIST and miR-23a, more *in vitro* studies have been performed. As the first step, we transfected miR-23a NC, miR-23a mimics or miR-23a inhibitors into DU145 and LNCAP cells. QRT-PCR was performed to verify the efficiency of transfection (Figure 3D). Then we detected the expression level of XIST in DU145 and LNCAP cells transfected with miR-23a mimics, miR-23a inhibitors or miR-23a NC. As the results suggested that, over-expression of miR-23a could not decrease the expression of XIST in either DU145 or LNNCAP cells (Figure 3E). On the other hand, over-expression of XIST significantly reduced the expression of miR-23a compared with control group (Figure 3F).

**Figure 4:**
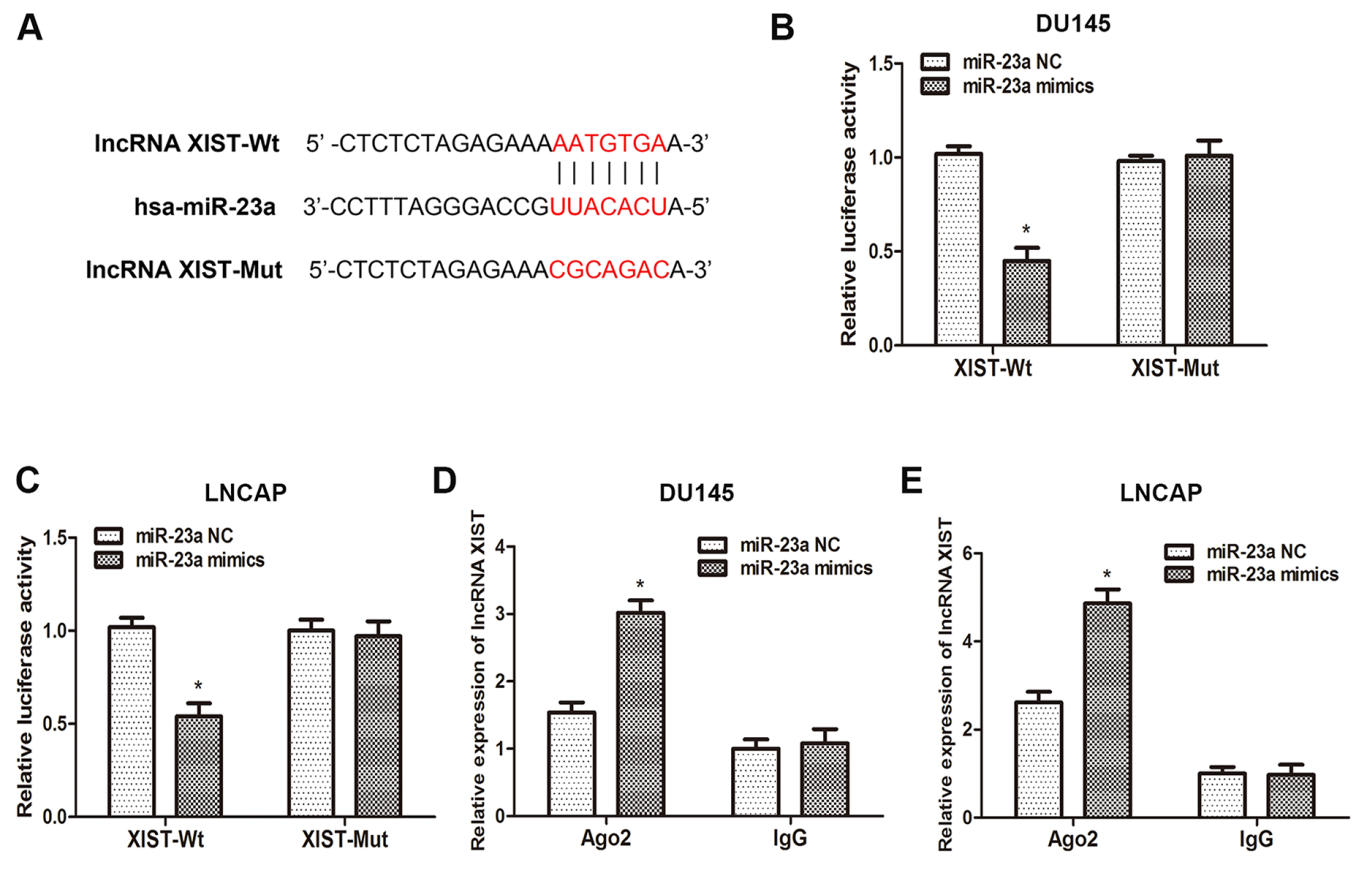
XIST inhibits miR-23a expression by directly targeting it **(A)** Putative binding site of miR-23a on XIST. **(B, C)** Dual-luciferase reporter assays were performed to determine luciferase activity in DU145 and LNCAP cells co-transfected with miR-23a mimics and XIST-Wt or XIST-Mut. *P<0.05 vs. miR-23a NC group. **(D, E)** RNA-IP assays were performed in DU145 and LNCAP cells transfected with miR-23a mimics and miR-23a NC. The expression of XIST was determined by qRT-PCR. *P<0.05 vs. miR-23a NC group. Data are presented as mean ± SD in three independent experiments.

To validate that lncRNA XIST directly binds to miR-23a, we made an in silico prediction of target sites in the sequence of miR-23a by using the Starbase v2.0 database (Figure 4A). Then, luciferase reporters including wild-type (XIST-WT) or mutated (XIST-Mut) miR-23a binding sites in XIST were constructed according to the prediction. As the results of the dual-luciferase reporter assay showed, luciferase activity was remarkably decreased in cells co-transfected with XIST-Wt and miR-23a mimics, but was not affected in cells co-transfected with XIST-Mut and miR-23a mimics (Figure 4B, 4C). Previous studies have demonstrated that microRNAs degrade RNA or repress translation via an Ago2-dependent pathway. Therefore, we employed an anti-Ago2 RIP assay in DU145 and LNCAP cells transfected with miR-23a mimics. As the data indicated, endogenous XIST was pulled down specifically in miR-23a overexpressed cells compared with control group, suggesting that miR-23a is a direct inhibitory target of lncRNA XIST. In sum, those data demonstrated that XIST regulated the expression of miR-23a by directly binding to it, but miR-23a could not induce the degradation of XIST in return.

### RKIP is a target gene of miR-23a and is regulated by XIST

**Figure 5:**
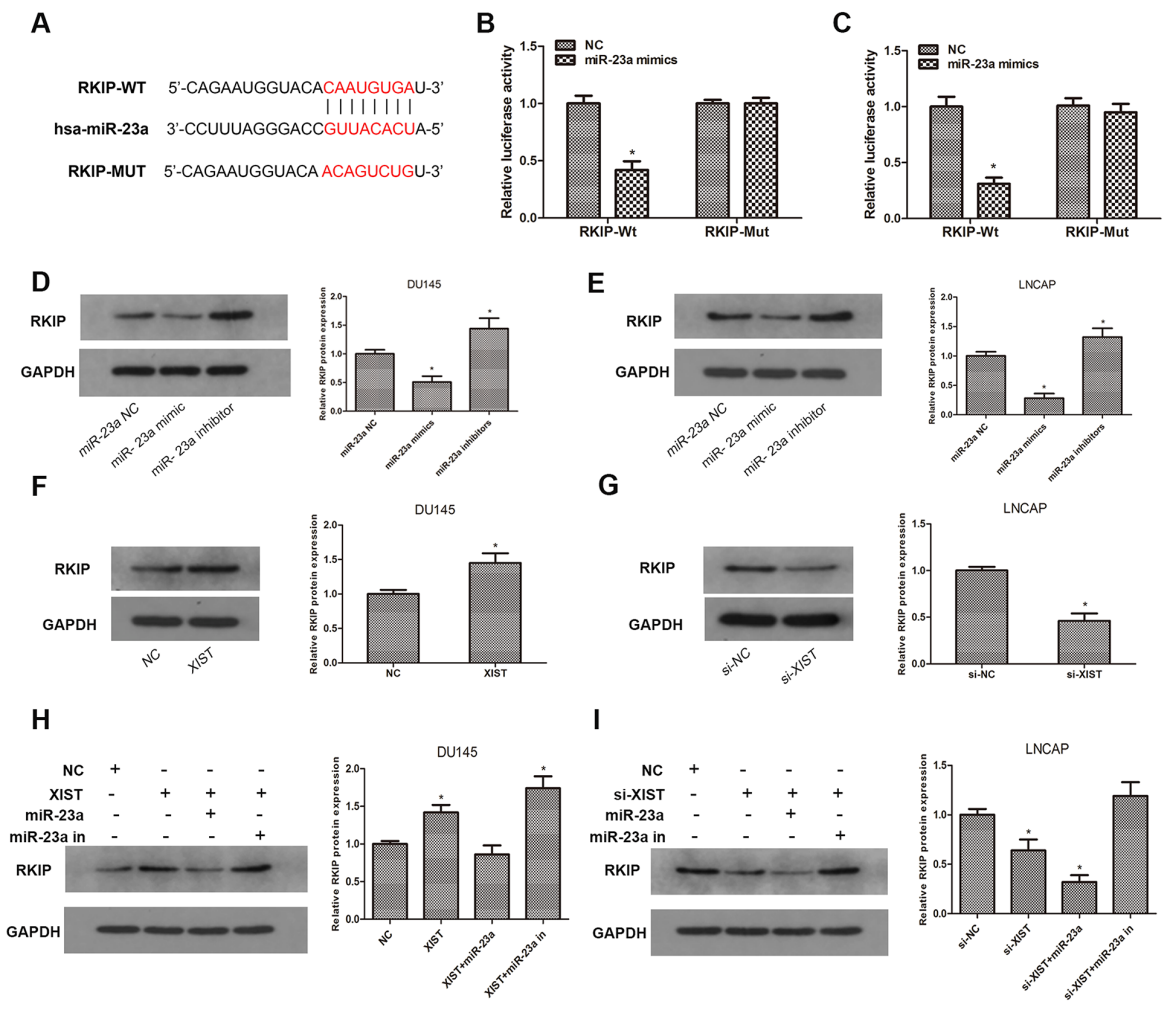
RKIP is a target gene of miR-23a and is regulated by XIST **(A)** Predicted miR-23a binding sites in the 3′UTR of RKIP (RKIP-Wt) and mutant sequence (RKIP-Mut) was shown. **(B, C)** Luciferase reporter assays were performed to determine luciferase activity in DU145 and LNCAP cells co-transfected with miR-23a mimics and luciferase reporters containing 3′UTR sequence of RKIP-Wt or RKIP-Mut. *P<0.05 vs. miR-23a NC group. **(D, E)** Relative expression of RKIP was examined by western blot in DU145 and LNCAP cells transfected with miR-23a mimics and inhibitors. *P<0.05 vs. miR-NC group. **(F, G)** Relative expression of RKIP in DU145 cells transfected with XIST or NC and LNCAP cells transfected with si-XIST or si-NC. *P<0.05 vs. NC group or si-NC group. **(H)** Relative expression of RKIP in DU145 cells co-transfected with XIST and miR-23a mimics or miR-23a inhibitors. *P<0.05 vs. NC group. **(I)** Relative expression of RKIP in LNCAP cells co-transfected with si-XIST and miR-23a or miR-23a inhibitors. *P<0.05 vs. si-NC group. Data are presented as mean ± SD from three separated experiments.

Previous studies have demonstrated that RKIP acts as a critical tumor suppressor in prostate cancer, and miR-23a has been reported to be inversely correlated with RKIP expression in a variety of human malignancies [20, 21]. To confirm whether miR-23a is involved in the regulation of RKIP in prostate cancer, we explored the TargetScan database and predicted that miR-23a may directly bind to RKIP in its 3′UTR (Figure 5A). To verify our prediction, we constructed luciferase reporter plasmids containing wild-type 3′UTR sequence of RKIP or mutant 3′UTR sequence. A luciferase reporter assay was performed after transfection with luciferase reporter plasmids and miR-23a mimics. As our data showed, luciferase activity in RKIP-Wt group transfected with miR-23a mimics was significantly inhibited compared with miR-23a NC, while there was no change in RKIP-Mut group (Figure 5B, 5C). To validate the prediction at the protein level, we examined the expression of RKIP by immunoblotting after miR-23a over-expression or knockdown. Our findings confirmed that knockdown of miR-23a led to a remarkable increase in the expression of RKIP, while over-expression of miR-23a caused a significant reduction of RKIP in contrast (Figure 5D, 5E). Taken together, these results indicated that miR-23a negatively regulated RKIP expression in prostate cancer cells by directly targeting the 3′UTR of RKIP.

To further investigate whether XIST functions as a ceRNA in the regulation of RKIP, we employed immunoblotting assays to determine the protein expression of RKIP after cells were transfected with XIST or si-XIST and miR-23a mimics or inhibitors. We found that overexpression of XIST markedly promoted the expression of RKIP, whereas miR-23a abrogated such increase in RKIP expression induced by XIST (Figure 5F, 5H). On the other hand, knockdown of XIST led to a reduction in RKIP, while which could be rescued by miR-23a inhibition (Figure 5G, 5I). These findings suggested that lncRNA XIST regulated the expression of RKIP in a ceRNA manner and miR-23a played a key role in XIST-mediated regulatory pathway.

### LncRNA XIST suppresses tumor growth of prostate cancer *in vivo*

**Figure 6:**
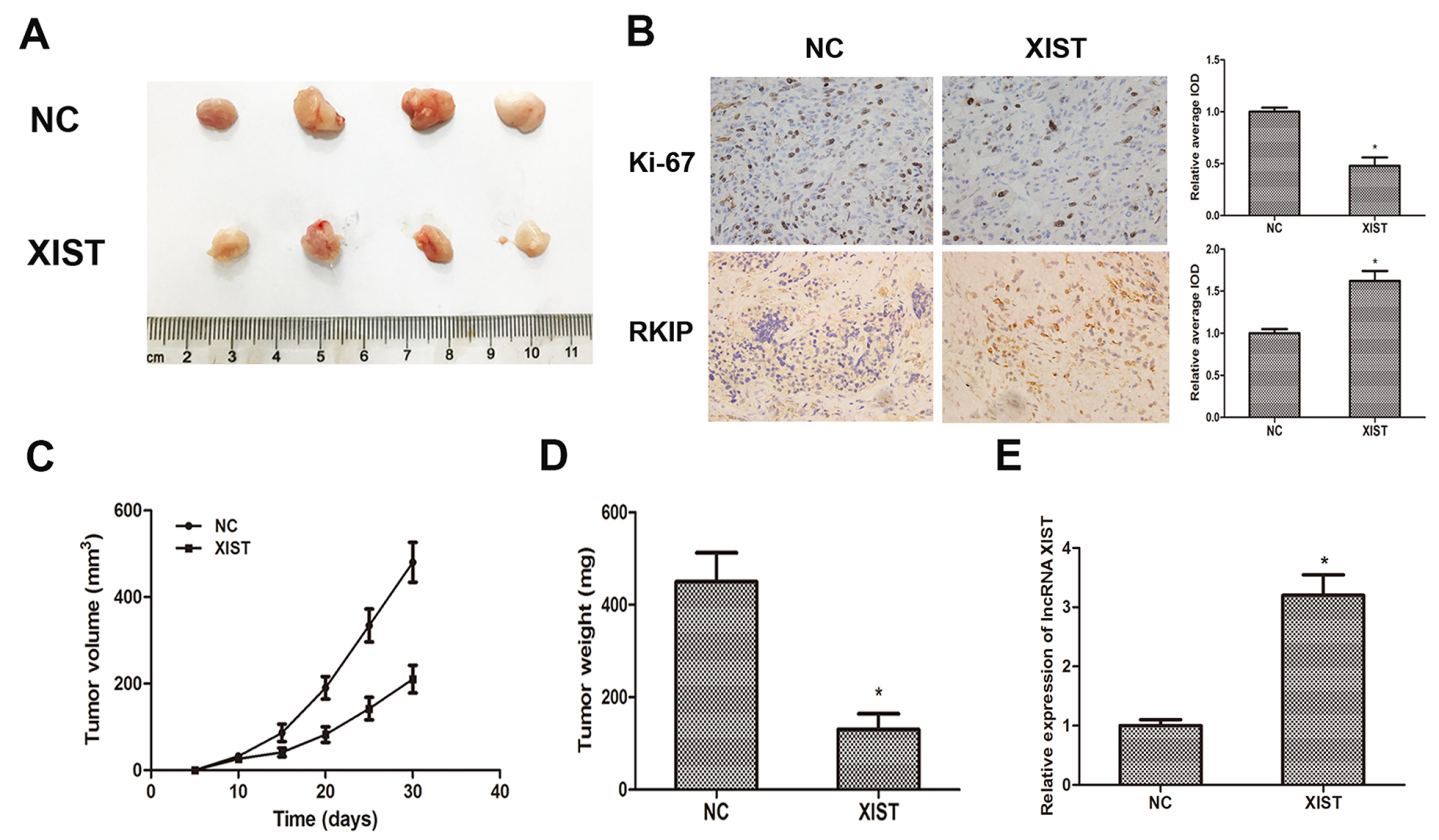
LncRNA XIST suppresses tumor growth of prostate cancer *in vivo* **(A)** Photographs of tumors excised from nude mice derived 28 days after subcutaneous inoculation of DU145 cells transfected with XIST or NC. **(B)** The expression of Ki-67 and RKIP in xenograft samples were assessed by Immunohistochemical staining. Analyses of relative average integrated optical density (IOD) using Image Pro-Plus software. **(C)** Tumor volumes were calculated every 5 days from day 5 to day 28 after inoculation. Bars indicate SD. **(D)** Tumor weight of xenograft tumors were measured and analyzed. *P<0.05 vs. NC group. **(E)** Qrt-PCR was performed to determine the expression of XIST in xenografts. *P<0.05 vs. NC group. Data are presented as mean ± SD in three independent experiments.

To further investigate whether XIST inhibits tumor growth *in vivo*, DU145 cells transfected with XIST or NC were inoculated into male nude mice. Xenograft tumors were examined 4 weeks after inoculation. PCR was performed to determine the expression of XIST, and the result showed that the expression of XIST was markedly increased in XIST overexpressing group as we expected (Figure 5E). Meanwhile, we found that tumor volumes and weights in XIST group were significantly less than those in NC group (Figure 6A, 6C, 6D). Furthermore, we detected the expression of Ki-67 and RKIP in xenograft samples via immunohistochemical (IHC) staining (Figure 6B). As the results showed, the number of Ki-67 positive cells in XIST group was obviously fewer than NC group, while RKIP expression was moderately increased in XIST group compared to NC group. These data revealed that lncRNA XIST could suppress tumorigenesis of prostate cancer *in vivo*.

## DISCUSSION

Prostate cancer is a heterogeneous type of malignancy with a high incidence in males. Based on the fact that most tumor cells respond well to ADT therapy in the initial stage, the application of novel anti-androgens has prominently improved life expectancy and alleviated suffering in patients [22]. Although localized tumors can be cured radically by surgery and hormonal therapy, it remains difficult to diagnose tumors early and prevent them from being castration-resistant. Due to the rapid development of basic medical research, the molecular mechanisms underlying the tumorigenesis and pathogenesis of prostate cancer have been uncovered gradually over past few years. A growing body of literature suggests that dysregulation of lncRNAs may contribute to tumor growth and progression [23–25]. Thus, exploring the role and biological functions of lncRNAs in prostate cancer will provide us new possibilities for diagnostics and treatment in clinical settings.

LncRNA XIST is located on the X chromosome in the X-inactivation center [26]. It was first identified as an oncogene in human glioblastoma stem cells and plays an important role in cell proliferation, migration and invasion in glioblastoma [27]. Recent studies have indicated that XIST was aberrantly expressed in several cancers, including gastric cancer, lung cancer, pancreatic cancer, hepatocellular carcinoma and osteosarcoma [28–32]. In the present study, we found that the expression of XIST in prostate cancer tissues was remarkably decreased compared with adjacent normal tissues. Low XIST expression levels were also correlated with high Gleason score, clinical stage and metastasis in patients with prostate cancer. Furthermore, in gain and loss of function assays, we verified that overexpressing XIST induced significant suppression in cell proliferation, cell migration and invasion ability of prostate cancer cells both *in vitro* and *in vivo*. On the other hand, knockdown of XIST led to an inverse result. These data suggest that lncRNA XIST may function as a tumor suppressor in prostate cancer.

Recent studies have reported that lncRNAs exhibit the ability to act as a miRNA sponge and modulate the derepression of miRNA targets at post-transcriptional level [33, 34]. Based on this theory, lncRNAs can antagonize the inhibitory effect of miRNA by competitively binding to it. For example, lncRNA H19 promoted colorectal cancer progression by epigenetically repressing miR-200a [35], and LncRNA UCA1 contributed to tumor growth by down-regulating miR-16 in bladder cancer [36]. LncRNA Gas5 inhibits carcinogenesis in human glioma by targeting miR-222 [37]. In our present study, we confirmed that miR-23a was highly expressed in prostate cancer samples and inversely associated with the expression of XIST. By using bioinformatics prediction, we found miR-23a may serves as a direct target of XIST. To determine whether XIST directly binds to miR-23a, luciferase reporter assays and RNA pull down assay were performed. In luciferase reporter assay, we confirmed the direct binding site between XIST and miR-23a. In RNA-IP assay, we found that XIST was significantly pulled down in miR-23a overexpressing cells. Collectively, our data suggest that XIST regulates miR-23a expression by directly binding to it.

Raf kinase inhibitor protein (RKIP) belongs to the phosphatidylethanolamine binding protein (PEBP) family and competitively inhibits Raf-mediated activation of mitogen-activated protein kinase/extracellular signal-regulated kinase (MAPK/ERK) [38, 39]. RKIP has been found to be expressed in a variety of organs, such as lung, liver and prostate [40–42]. It also has been reported that low expression level of RKIP may be correlated with several human malignancies. For example, Hagan et al showed that RKIP was markedly reduced in metastatic breast cancer [43]. Mulla et al found a reduction in RKIP related to poor prognosis in colorectal cancer patients [44]. Fu et al has also demonstrated that RKIP functions as a tumor suppressor in prostate cancer [20]. A recent study suggested that lncRNA GNAT1-1 inhibited tumorigenesis and metastasis in colorectal cancer by regulating the RKIP-NF-κB signaling pathway [45]. However, the interaction between long non-coding RNA and RKIP in prostate cancer has not been elucidated. In the present study, we verified that XIST could positively regulate the expression of RKIP post-transcriptionally through sponging miR-23a in prostate cancer cells. In contrast, overexpressed miR-23a did not change the expression level of XIST, but abrogated the up-regulation of RKIP induced by XIST overexpression. Taken all together, these results suggested that lncRNA XIST functioned as a ceRNA, and this positive regulatory effect of XIST on RKIP required the inhibition of miR-23a.

In conclusion, our study revealed that lncRNA XIST was markedly decreased in prostate cancer specimens and functioned as a tumor suppressor, which played a key role in regulating malignancies in prostate cancer. Additionally, we determined that lncRNA XIST positively regulated RKIP expression at post-transcription level by targeting miR-23a in prostate cancer. Therefore, our study indicated that lncRNA XIST has a potential to be applied as a novel therapeutic target into the treatment of prostate cancer.

## MATERIALS AND METHODS

### Clinical specimens

A total of 62 paired prostate cancer tissues and adjacent normal tissues were obtained from patients who had undergone surgery in the Department of Urology, Ren’min Hospital of Wuhan University from 2010-2012. All protocols in this study were approved by the Institutional Ethics Committee of Ren’min Hospital of Wuhan University. Informed written consents were signed before operation and relevant clinical information was gathered from patients. All tissue samples were frozen immediately in liquid nitrogen and then stored at -80°C until RNA extraction. Tumors were classified according to the TNM staging system, preoperative PSA level and Gleason score. All clinic pathological information of patients has been listed in Table 1.

### Cell lines and cell culture

Three prostate cancer cell lines (PC3, DU145 and LNCAP) and a human prostate epithelial cell line (RWPE1) were purchased from the ATCC (American Type Culture Collection, Manassas, VA). Cells were cultured in RPMI 1640 medium supplemented with 10% fetal bovine serum (GIBCO, MA, USA) at 37°C with 5% CO_2_.

### Plasmid construction and transfection

Full-length XIST from RWPE1 cells was amplified by PCR and then cloned into pDC315 vector (Microbix, CA). Recombinant adenoviruses were produced using HEK-293T packaging cells. 48 h later, adenoviruses over-expressing XIST were harvested and purified. The infection efficiency of the adenovirus in our present study was nearly 100%. The full sequence of human RKIP 3′UTR containing putative miR-23a binding sites was also amplified and then cloned into psiCHECK-2 vector (Promega, WI, USA). Si-XIST (si-XIST sense: 5′-GGCCTGTTATGTGTGTGATTATATT-3′), miR-23a mimics, miR-23a inhibitor and miR-23a negative control were purchased from Biossci Company (Wuhan, China). All transfections were performed using a Lipofectamine 2000 kit (Invitrogen, USA), following the manufacturer’s instructions.

### Quantitative real-time PCR

Total RNA was isolated from tumor specimens and cancer cell lines using Trizol reagent (Invitrogen, USA). The purity of RNA was examined by spectrophotometry and the first strand cDNA was synthesized using reverse transcription Reagents (ABI, CA) or the TaqManH MicroRNA Reverse Transcription Kit (ABI, CA) following the manufacturer’s instructions. QRT-PCR was performed using SYBRH Select Master Mix for CFX (Invitrogen) and using the CFX Connet TM real-time PCR system (Bio-Rad, USA). All results were normalized to the expression of GAPDH or snRNA U6. The quantitative analysis was calculated by using 2^-ΔΔCt^ method. All the primers are shown in Table 2.

**Table 2:** Primer sequences used for qRT-PCR

GENE	Primer sequences (5′-3′)
lncRNA XIST	F: CTCTCCATTGGGTTCAC
	R: GCGGCAGGTCTTAAGAGATGAG
RKIP	F:CAATGACATCAGCAGTGGCACAGTC
	R: CACAAGTCATCCCACTCGGCCTGAC
miR-23a	F: CCAGCTACACTGGGCAGCAGCAATTCATGTTT
	R: CTCAACTGGTGTCGTGGA
U6 snRNA	F: GCUUCGGCAGCACAUAUACUAAAAU
	R: CGCUUCACGAAUUUGCGUGUCAU
GAPDH	F: TGAAGGTCGGTGTGAACGGATTTGGTC
	R: CATGTAGGCCATGAGGTCCACCAC

### Cell proliferation assay

Cell proliferation was measured using a Cell Counting Kit-8 (Beyotime Institute of Bio-technology, Jiangsu, China). After being inoculated into 96-well plates at a density of 2 × 10^3^ cells/well, cells were stained with 20 μL of CCK8 reagent 48 hours after transfection. 2 hours after incubation, cell viability was measured by detecting the absorbance of samples at 450 nm.

### Cell cycle assay

Cell proliferation was investigated by flow cytometry analysis. Cells were harvested and fixed in 70% ethanol overnight. Then, cells were added with RNase (50 μg/mL) and PI (50μg/mL) (BD, USA). After incubation for 30min, samples were subjected to flow cytometer (FACScan, BD, USA) for analysis. Data were analyzed using CELL Quest 3.0 software.

### Wound healing assay

Cells were seeded in 6-well plates and incubated in complete medium for 12 hours. Wounds were generated using a 200 μL pipette tip. After replacing the supernatant with serum-free medium, cells were cultured for 48 hours. The wound gaps were photographed at different time points using a microscopy (Olympus, Japan).

### Cell invasion assays

In this study, we used a 24-well Transwell chamber (Corning, MA, USA). Matrigel (BD, USA) was smeared on the upper chambers before experiments. In brief, Cells (3 × 10^4^ cells in 200 μL serum free medium) were seeded into the upper Matrigel-coated chambers. Medium containing 10% FBS was placed in the lower chamber. At 24 hours after incubation, cells on the upper chamber membrane were wiped away with a cotton swab. Cells on the lower chamber membrane were fixed with methanol, stained with 0.1% crystal violet, counted in five random scopes and photographed.

### Luciferase reporter assays

XIST fragment containing putative or mutated miR-23a binding site was amplified by PCR and then cloned into a pmirGLO Dual-luciferase miRNA Target Expression Vector (Promega, WI, USA). The recombinant reporter vector was named as XIST-wild-type (XIST-Wt) or XIST-mutated-type (XIST-Mut). XIST-Wt or XIST-mut was co-transfected with miR-23a NC or miR-23a mimics using Lipofectamine 2000 (Invitrogen, USA). Luciferase assay was performed 48 h after transfection using a Dual-Luciferase Reporter Assay System (Promega, WI, USA).

Similar to above, the putative and mutated miR-23a target binding sequence in RKIP were synthesized and cloned into luciferase reporter to generate the wild-type (RKIP-Wt) or mutated-type (RKIP-Mut) reporter plasmids. The transfection procedure was the same as described previously.

### RNA immunoprecipitation assay

RNA immunoprecipitation assay was performed using the EZ-Magna RIP RNA-binding protein immunoprecipitation kit (Millipore, MA, USA). All procedures were implemented following manufacturer’s instructions. PC3 and DU145 cells were lysed with RIP lysis buffer with RNase inhibitor, and subsequently incubated with RIP buffer. Antibody against argonaute2 (Ago2) (Millipore) was used to form conjugated magnetic beads. XIST fold enrichment of RNA immunoprecipitation was normalized to the RIP fraction of control antibody IgG (Millipore), and was examined by qRT-PCR analysis.

### Western blot analysis

Cells were collected and lysed using RIPA buffer with PMSF (Beyotime, Beijing, China) on ice. Protein concentration was qualified using a bicinchoninic acid assay (BCA) kit (Beyotime, Beijing, China). Equivalent amounts of protein samples were separated by 10% sodium dodecyl sulfate-polyacrylamide (SDS-PAGE) gels electrophoresis, and transferred to (polyvinylidene fluoride) PVDF membranes subsequently. Membranes were blocked in Tris-buffered saline (TBS) containing 5% nonfat milk. After that, membranes were incubated with primary antibody against RKIP (#13006, CST, USA) and GAPDH (#2118S, CST, USA) at 4°C overnight, followed by incubation with secondary antibodies, detected by enhanced chemiluminescent (ECL) and qualified using ImageJ software (NIH, MD, USA).

### Tumor xenograft formation assay

All BALB/C nude mice (6 weeks old, male) were purchased from the Animal Center of Ren’min Hospital of Wuhan University and maintained in pathogen free facilities. All procedures were performed in accordance with the National Institutes of Health Guide for the Care and Use of Laboratory Animals, and approved by the Animal Care and Use Committee of Wuhan University. DU145 Cells (3 × 10^6^, 200 μL) transfected with XIST or negative control were subcutaneously injected into the left flank of nude mice. Tumor growth was examined every 5 days, and tumor volume was calculated using the equation: Volume = (length × width^2^) / 2(mm^3^). All mice were sacrificed 4 weeks after inoculation, and tumors were excised and photographed.

### Immunohistochemistry

Tissues from xenograft formation assay were fixed in 4% paraformaldehyde, embedded in paraffin and then cut in 4.5 μm thickness. The primary antibodies against Ki-67 (Santa Cruz, USA) and RKIP (#13006, CST, USA) were used at a dilution of 1:200. Ki-67 kit was used to evaluate the proliferation of tumors. For quantitation, average integrated optical density (IOD) was obtained by analyzing five fields in each slide evaluated by Image-Pro Plus software (ver 6.0) for immunohistochemical staining of Ki-67 and RKIP. The average IOD of tumor tissue was divided by the average IOD of paired normal tissue to get the relative average IOD. All sections were photographed at a magnification of × 400.

### Statistical analysis

Data analysis was performed using SPSS20.0 (SPSS, Chicago, USA). All data were represented as means ± standard deviation (SD). The significance of differences between groups were assessed by Student’s t test and χ2 test as appropriate. P < 0.05 was considered as statistically significant.
